# Local Conditions Influence the Prokaryotic Communities Associated With the Mesophotic Black Coral *Antipathella subpinnata*

**DOI:** 10.3389/fmicb.2020.537813

**Published:** 2020-10-06

**Authors:** Jeroen A. J. M. van de Water, Martina Coppari, Francesco Enrichetti, Christine Ferrier-Pagès, Marzia Bo

**Affiliations:** ^1^Centre Scientifique de Monaco, Monaco, Monaco; ^2^Dipartimento di Scienze della Terra, dell’Ambiente e della Vita, Università degli Studi di Genova, Genova, Italy; ^3^Consorzio Nazionale Interuniversitario per le Scienze del Mare, Rome, Italy

**Keywords:** 16S rRNA gene amplicon analysis, black coral, microbiome, gorgonian, mesophotic coral, *Antipathella subpinnata*, microbiome flexibility, phylosymbiosis

## Abstract

Black corals are important habitat-forming species in the mesophotic and deep-sea zones of the world’s oceans because of their arborescent colony structure and tendency to form animal forests. Although we have started unraveling the ecology of mesophotic black corals, the importance of the associated microbes to their health has remained unexplored. Here, we provide in-depth assessments of black coral-microbe symbioses by investigating the spatial and temporal stability of these associations, and make comparisons with a sympatric octocoral with similar colony structure. To this end, we collected samples of *Antipathella subpinnata* colonies from three mesophotic shoals situated along the Ligurian Coast of the Mediterranean Sea (Bordighera, Portofino, Savona) in the spring of 2017. At the Portofino shoal, samples of *A. subpinnata* and the gorgonian *Eunicella cavolini* were collected in November 2016 and May 2017. Bacterial communities were profiled using 16S rRNA gene amplicon sequencing. The bacterial community of *E. cavolini* was consistently dominated by *Endozoicomonas*. Contrastingly, the black coral microbiome was more diverse, and was primarily composed of numerous Bacteroidetes, Alpha- and Gammaproteobacterial taxa, putatively involved in all steps of the nitrogen and sulfur cycles. Compositional differences in the *A. subpinnata* microbiome existed between all locations and both time points, and no phylotypes were consistently associated with *A. subpinnata*. This highlights that local conditions may influence the bacterial community structure and potentially nutrient cycling within the *A. subpinnata* holobiont. But it also suggests that this coral holobiont possesses a high degree of microbiome flexibility, which may be a mechanism to acclimate to environmental change.

## Introduction

Antipatharians, commonly known as black corals, can be found throughout the world’s oceans from tropical to polar latitudes (Wagner et al., 2012). This taxon of hexacorals consists of about 247 species (Brugler and France, 2007) and is particularly diverse in tropical and subtropical regions (Tazioli et al., 2007; Wagner et al., 2012). Dense assemblages of black corals have been reported in the mesophotic (40–150 m depth) (Cairns, 2007; Bo et al., 2019) and deep-sea zones (Molodtsova and Opresko, 2017), where they provide critically important habitat. Because of their typically wide bathymetric distribution, it has been difficult to collect specimens and make direct observations of black corals. As such, many aspects of the black coral ecology and biology remain unknown. However, access to remotely operated vehicles and technical diving now allows us to explore black coral communities and investigate the physiology and microbial ecology of these organisms.

Macro-organisms live in symbioses with microbes (an assemblage termed the ‘holobiont’), which are known to play important roles in host health, and contribute to their host’s adaptation and acclimation to environmental change (Bosch and McFall-Ngai, 2011; McFall-Ngai et al., 2013). In corals, mutualist and commensal bacteria provide the host with nutrients (Benavides et al., 2017) and protect it against pathogens through occupation of available niches (Rohwer et al., 2002; Rypien et al., 2010) and the secretion of antimicrobial compounds (Ritchie, 2006; Nissimov et al., 2009; Kvennefors et al., 2012; Shnit-Orland et al., 2012). For the fitness of the holobiont, it is therefore important to maintain a microbial community with a stable repertoire of metabolic and protective functions. In fact, signals of phylosymbiosis in the coral holobiont have shown that there is a long-term evolutionary history between corals and various microbial taxa (Pollock et al., 2018; van de Water et al., 2018b). The coral microbiome has been extensively studied in tropical and temperate scleractinian corals (hexacorals) and Mediterranean gorgonians (octocorals) (reviewed in Bourne et al., 2007; Hernandez-Agreda et al., 2017; van de Water et al., 2018a, respectively). While the microbial community compositions of the latter have been found to be stable on both spatial and temporal scales (van de Water et al., 2016, 2017, 2018b), the microbiota of scleractinians tends to be more diverse and variable (Littman et al., 2009; Hernandez-Agreda et al., 2016; Pollock et al., 2018). It should, however, be noted that in contrast to Mediterranean octocorals, the majority of scleractinian corals studied are in an intricate symbiosis with Symbiodiniaceae and that these algal symbionts are known to affect the microbiota of marine invertebrates (Bourne et al., 2013). Generally, however, species-specific microbes and environmentally responsive microbes can be found within the holobiont of each coral species (Hernandez-Agreda et al., 2016; van de Water et al., 2017; Pollock et al., 2018), with differences observed between the microbiota in coral tissues and other compartments, such as mucus and skeleton (Sweet et al., 2011; Apprill et al., 2016; Pollock et al., 2018; Bednarz et al., 2019). Although of high ecological importance in the mesophotic zone and deep sea, the microbial ecology of black corals has so far received little attention.

Initial investigations established cultures of various microbes associated with black corals, particularly Firmicutes and Actinobacteria from *Antipathes dichotoma* (Pallas, 1766) (Zhang et al., 2012), and a range of Gammaproteobacteria and Actinobacteria from *Stichopathes luetkeni* (Santiago-Vázquez et al., 2007). However, as most microbes cannot be cultured yet, a culture-dependent approach may not provide the best overview of the black coral-associated microbiota. Early culture-independent approaches found primarily Rhodobacterales and Pseudomonadales on an unidentified deep-sea black coral (Penn et al., 2006), but a more diverse microbiota associated with *S. luetkeni*, consisting mainly of a range of Proteobacteria, Actinobacteria, Firmicutes, Cytophaga-Flavobacterium and Chloroflexi (Santiago-Vázquez et al., 2007). Two recent studies using 16S rRNA gene amplicon sequencing on the widely distributed deep-sea black coral *Leiopathes glaberrima* (Esper, 1788) and two Pacific *Antipathes* spp. found a similar microbial community composition at the phylum level (Dannenberg, 2016; Liu et al., 2018). However, significant differences in the microbiota were observed between sampling locations. Interestingly, only one phylotype, which belonged to the genus *Endozoicomonas*, was present in all colonies of *L. glaberrima* (Dannenberg, 2016). These bacteria are commonly present in the microbiota of scleractinian corals, gorgonians and various other marine invertebrates, where they are believed to be important for host health (Neave et al., 2016).

Black corals are also associated with various eukaryotic microbes. For example, several Hawaiian (Wagner et al., 2011) and Indonesian (Bo et al., 2011a) species have been found to engage in symbioses with unicellular algae of the genus Symbiodiniaceae. These algae contribute significantly to the nutrition of shallow reef-building scleractinian corals, but their relevance to black corals in the non-photic zone remains unclear. Using a transcriptomics approach, apicomplexans were found to be part of the *L. glaberrima* holobiont as well (Dannenberg, 2016). Further genomic characterization of these corallicolid apicomplexans revealed that members of this diverse clade are widespread associates of deep-sea corals. Although their ecological role remains uncertain, they do possess some characteristics of a parasitic lifestyle (Vohsen et al., 2020).

While these studies have provided initial assessments of the microbiota of black corals, a comprehensive assessment of the black coral-associated microbial communities is an important first step to understand the ecological success of these animals better. *Antipathella subpinnata* (Ellis and Solander, 1786) is the most common mesophotic black coral species in the Mediterranean Sea, but it has also been reported in the Atlantic Ocean (OCEANA, 2011; de Matos et al., 2014). Its arborescent colonies of up to 1.5 m form large (Bo et al., 2018) and dense (5.2 colonies m^–2^) aggregations (Bo et al., 2009; de Matos et al., 2014) on hard substrates, including shipwrecks, between 55 and 500 m (Bo et al., 2008, 2012b; Mastrototaro et al., 2010). This makes *A. subpinnata* one of the most important habitat-forming species that provides structural complexity to the Mediterranean mesophotic ecosystems and serves as a refuge for a rich associated fauna, including species of economic importance, such as seabream, jack mackerel, catshark and octopus (personal observations, Bo et al., 2008, 2009; Cau et al., 2017). Unfortunately, commercial fishing activities and entanglements by lost fishing lines or nets have caused significant damage to local populations of *A. subpinnata* (Bo et al., 2014; Oliveira et al., 2015). While several aspects of *A. subpinnata*’s reproductive biology (Gaino and Scoccia, 2010; Coppari et al., 2019) and ecology, including its interactions with various macro-epibionts (Bo et al., 2011b; Gaino et al., 2013) and feeding strategies (Coppari et al., 2020), have been studied, its microbial associates have never been investigated.

*Antipathella subpinnata* commonly lives in mixed assemblages with arborescent gorgonian octocorals, such as *Paramuricea clavata* (Risso, 1826) (Enrichetti, 2019; Chimienti et al., 2020) and *Eunicella cavolini* (Koch, 1887) (Bo et al., 2009; Gori et al., 2017), which are commonly found at shallow and mesophotic depths. While it is unknown how these black corals and gorgonians interact, the similar colony structures and polyps sizes (Weinberg, 1976; Bo et al., 2018) suggest that they may occupy the same trophic niche. Comparison of the natural ^13^C and ^15^N stable isotope signatures of the tissues of these corals indicates that this may indeed be the case (Gori et al., 2017; Coppari et al., 2020). *E. cavolini* may thus be a competitor for food resources of *A. subpinnata*, particularly, because the polyp density in *E. cavolini* is ∼3-fold higher compared with *A. subpinnata* (Weinberg, 1976; Bo et al., 2018). Besides, this gorgonian has been shown to be a strong competitor in physical interactions with other gorgonians (Turicchia et al., 2020). In contrast to *A. subpinnata*, the prokaryotic communities associated with shallow *E. cavolini* colonies have been previously described in detail (van de Water et al., 2017, 2018b), showing a relatively stable microbiota dominated by a few bacterial phylotypes, in particular *Endozoicomonas*. A comparison of the microbiota of these two distantly related but sympatric species in the mesophotic zone may provide insights into common microbial taxa, possibly linked to the trophic niche or the mesophotic environment.

Here, we describe the spatial and temporal patterns in the bacterial communities of the arborescent black coral *A. subpinnata*. In addition, we provide a comparison with the gorgonian *E. cavolini* to assess differences between the microbiota of these two sympatric species. We show that, in contrast to the gorgonian, the bacterial communities of *A. subpinnata* are highly diverse and differ significantly between locations and between autumn and spring time points. Interestingly, no bacteria were consistently associated with this species across space and time, suggesting that local environmental conditions are the main drivers of the microbiota associated with this black coral.

## Materials and Methods

### Study Sites and Sampling Procedures

**FIGURE 1:**
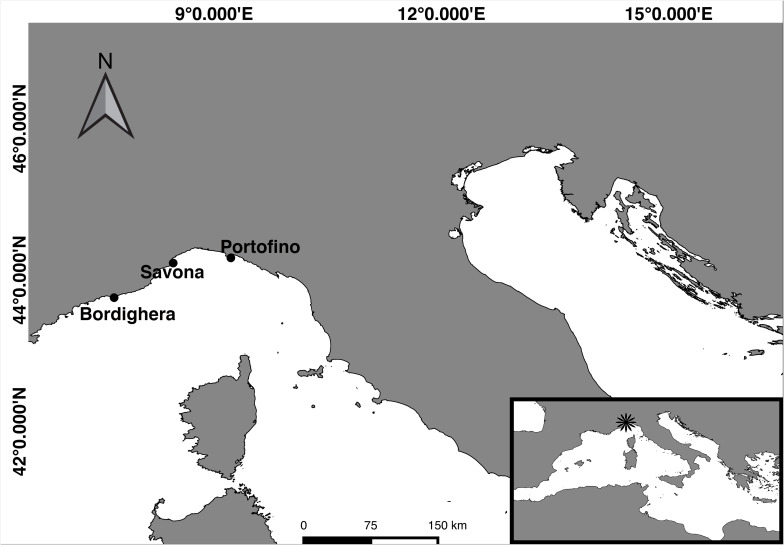
Map of the study sites along the Ligurian coast. Samples were collected from three mesophotic shoals near Bordighera, Savona, and Portofino.

To assess the effect of seasonality on the microbiome of *Antipathella subpinnata*, samples (one per colony) were collected by technical diving in Portofino (coordinates 44° 17.63′ N, 9° 13.27′ E, 67 m depth) (Figure 1) in autumn (*n* = 10 colonies, 30 November 2016, seawater temperature of 17.8°C) and spring (*n* = 10 colonies, 10 May 2017: seawater temperature 14.2°C). Here, samples of 6 colonies of *Eunicella cavolini* were also collected at both time points. To characterize the spatial stability in the microbiome of *A. subpinnata*, samples were collected from two other locations off the Ligurian coast near Bordighera (*n* = 10 colonies, coordinates 43° 46.11′ N, 7° 40.82′ E, 63 m depth, 21 April 2017, seawater temperature 14.1°C) and Savona (*n* = 12 colonies, coordinates 44° 13.52′ N, 8° 27.61′ E, 70 m depth, 15 May 2017, seawater temperature 14.3°C) (Figure 1) in spring 2017. Fragments of approximately 10 cm were cut from colonies using scissors and placed in a zip-lock bag. On board the vessel, samples were rinsed twice with 0.2 μm filtered seawater and stored in RNA*later* at 4°C until further processing. From each site and time point, three replicates of 2 L seawater were collected close to the sampling site (about 2–5 m above the black coral colonies) in a Niskin bottle. The collected seawater was sequentially filtered through 3 and 0.2 μm Whatman Nucleopore Track-Etched filters (Sigma-Aldrich) and the 0.2 μm filter was kept in RNA*later* at 4°C. An overview of the sample collection can be found in Table 1.

**TABLE 1:** Overview of sample collection and distances between sampling locations.

	**Portofino**		**Bordighera**	**Savona**
	**November 2016**	**May 2017**	**April 2017**	**May 2017**
*A. subpinnata*	10	10	10	12
*E. cavolini*	6	6		
Seawater	3	3	3	3
Distance between sampling locations	Portofino –Bordighera		∼135 km	
	Portofino – Savona		∼ 60 km	
	Bordighera – Savona		∼ 90 km	

**FIGURE 2:**
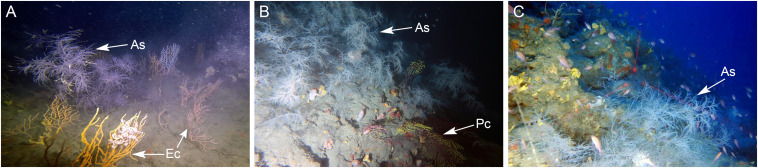
Populations of *Antipathella subpinnata* on the shoals near **(A)** Portofino at 67 m depth, **(B)** Bordighera at 63 m depth and **(C)** Savona at 70 m depth. Colonies of *A. subpinnata* (As) and the sympatric gorgonians *Paramuricea clavata* (Pc) and *Eunicella cavolini* (Ec) are indicated with arrows.

The three sites are characterized by rocky shoals with a maximum height of 10 m, which emerge from a gently sloping, sandy or detritic sea-bottom. The Portofino shoal is a large, isolated rocky shoal under strong current conditions. It is dominated by a dense forest of *Eunicella cavolini* and a small population of *A. subpinnata* is located on the steep westernmost side of the shoal (Figure 2A). The Bordighera shoal consists of scattered large rocky boulders, which were heavily silted and host a dense population of *A. subpinnata*, occasionally co-existing with the gorgonian *Paramuricea clavata* (Risso, 1826) on the margins of this animal forest (Figure 2B). The Savona shoal extends parallel to the coast and is a gently sloping, coralligenous elevation mainly hosting a scattered assemblage of gorgonians. At this site, *A. subpinnata* colonies can be found with a sparse distribution (Figure 2C). Additional information about the benthic communities and environmental conditions at these study sites can be found in Enrichetti et al. (2019).

### DNA Extraction and Sequencing Library Construction

To extract the DNA from both coral tissue and filter retentate, the DNeasy PowerBiofilm Kit (QIAGEN, Hilden, Germany) was used according to the manufacturer’s protocol, with the exception that the bead beating was performed at 30 Hz for 1 min using a CryoMill (Retsch, Haan, Germany) at room temperature. Extracted DNA was shipped to Macrogen (Seoul, Republic of Korea) for sequencing library construction using the 341F (5′-CCTACGGGNGGCWGCAG-3′) and 805R (5′-GACTACHVGGGTATCTAATCC-3′) primers that target the V3-V4 regions of the 16S rRNA gene (Klindworth et al., 2012), and 2x 300 bp paired-end sequencing (with a 30% PhiX control spike-in) on the Illumina MiSeq platform.

Negative DNA extraction kit controls (i.e., an extraction procedure without sample added) were not included in the sequencing run, although DNA extraction kits are known to be contaminated with microbial DNA. This may affect 16S rRNA gene amplicon sequencing results (Salter et al., 2014), especially in low bacterial biomass samples, whereas the impact will be low on high bacterial biomass samples (Glassing et al., 2016). To estimate the potential contamination in our DNA extractions, the amount of microbial DNA present in 2 μl of coral and seawater samples and negative DNA extraction kit controls was quantified using the Femto Bacterial DNA Quantification Kit (Zymo Research, Irvine, CA, United States) following the manufacturer’s protocol. Concentrations were 2.88 ± 0.92 ng/μl (*C*_*T*_ = 15.0 ± 0.23) and 0.148 ± 0.015 ng/μl (*C*_*T*_ = 19.6 ± 0.08) of bacterial DNA in seawater and coral samples, respectively. The negative DNA extraction kit controls contained 0.02 ± 0.05 pg/μl (*C*_*T*_ = 32.4 ± 1.28) of bacterial DNA. As such, the potential contamination was estimated to be low and had likely no to little impact on our results.

### 16S rRNA Gene Amplicon Data Processing and Analysis

The 16S rRNA gene amplicon data was analyzed using the UNOISE2 pipeline (Edgar, 2016) as implemented in the USEARCH package (version 9.2^1^) (Edgar, 2010). The raw forward (R1) and reverse (R2) sequence fastq files of the 66 samples contained a total of 20,259,044 reads (ranging between 183,112 and 386,636 reads per sample) with an average Q20 score of 89.43% and a Q30 score of 81.64%. R1 and R2 paired reads were merged using -fastq_mergepairs. Primer sequences were trimmed using -fastx_truncate and reads were quality filtered with the -fastq_filter script, generating a filtered fasta file containing 8,163,975 reads with an average length of 390 bp. Unique sequences were identified using the –fastx_uniques script followed by denoising of the sequence dataset with the UNOISE2 algorithm, obtaining 16,450 denoised sequences or ‘zero-radius OTUs’ (zOTU, Operational Taxonomic Unit). The –usearch_global script was then used to generate an OTU table at the 97% similarity level, containing 13,076 OTUs and an average 116,685 reads per sample (range 73,183 and 165,196 reads). The taxonomy was assigned to each OTU based on the SILVA database (release v123) (Quast et al., 2013) using the -sintax algorithm. The OTU table was converted to the HDF5 biom format and taxonomic assignment metadata was added.

Unassigned OTUs, and OTUs classified as chloroplast or mitochondria were excluded from the dataset. The most abundant OTU, OTU1 (representing 47% of all quality filtered reads), was also removed from the dataset after it was identified as a match to the mitochondrial 12S rRNA gene of black corals. [This high background amplification reduces the sequencing depth of the microbial community dramatically, and thereby could underestimate the diversity. Future studies on black coral microbiomes should take into account that in order to fully capture the microbial diversity of the black coral microbiome relatively deep sequencing is required. This result also shows that the 341F/805R primer set presents a strong amplification bias for the 12S rRNA gene of black corals. We therefore also recommend the testing of a range of primer pairs to assess which pair provides the least 12S rRNA gene amplification without compromising the accuracy of the assessment of the bacterial community composition.] Three *A. subpinnata* samples collected in spring 2017 were identified as outliers (two samples from Portofino and one sample from Bordighera) during sample quality assessment of the dataset during differential abundance analysis (see below), and were also removed from the OTU table.

Because negative DNA extraction kit controls had not been included in the sequencing run, a statistical approach was employed to identify and remove potential contaminant sequences as an additional quality control step. The ‘Frequency’ method of the R-package *decontam* (Davis et al., 2018) was used on (1) seawater and (2) coral (*A. subpinn*ata and *E. cavolini*) samples separately because of the large difference in microbial DNA concentration in the samples. In case of contaminants, a bimodal distribution of the decontam scores is expected (Davis et al., 2018), but this was not observed in both cases (Supplementary File S1). In addition, all OTUs within the low decontam score (*P*^∗^ < 0.05) range (1 and 24 OTUs in the seawater and coral datasets, respectively) had a very low prevalence (present in 2–3 samples), indicating low classification accuracy and sensitivity (Davis et al., 2018) (Supplementary File S1). Taken together, no potential contaminant sequences were identified in the dataset.

The final OTU table contained 5,621,675 reads belonging to 12,368 OTUs, with an average of 85,177 reads per sample (min 19,354, max 124,694). The unfiltered OTU table, sample metadata and representative sequences of each OTU are provided in the Supplementary Data S1–S3. Raw sequences were deposited in the NCBI Sequence Read Archive (SRA) under accession number PRJNA506661.

The OTU table was rarefied to 19,354 reads per sample, containing 9,330 OTUs. Alpha diversity metrics (richness: observed OTUs, diversity: Shannon-Wiener *H*, evenness: Simpson’s *E*) were calculated from the OTU table using QIIME v1.9 (Caporaso et al., 2010). The *phyloseq* package (McMurdie and Holmes, 2013) integrated in R was used to generate relative abundance plots and heatmaps. Using PRIMER 6 & PERMANOVA+ (PRIMER-E Ltd, Auckland, New Zealand) (Clarke and Gorley, 2006; Anderson and Walsh, 2013), Permutational Analysis of Variance (PERMANOVA) performed under Type III partial sums of squares and 9999 permutations under the reduced model was used to statistically assess differences in bacterial communities’ alpha and beta diversity between locations, time points and species. Principal Coordinates Analysis (PCoA) on square root-transformed Bray–Curtis similarity matrices was used to visualize these differences in beta diversity. Negative binomial generalized linear modeling and Wald tests for pair-wise comparisons as implemented in the *DESeq2* package (version 1.20.0) (Love et al., 2014) in R (version 3.5.0) (R Core Team, 2018), were used for differential abundance analysis to test which bacterial OTUs were differentially abundant (adjusted *p*-value < 0.01) between time points and sampling sites. Overall effects of location and time point on the coral-associated bacterial communities were investigated using the adonis() function in the R-package *vegan* (Oksanen et al., 2018). Core microbiome analyses were performed on the non-rarefied OTU table to identify the core microbiome (i.e., OTUs present in 80–100% of the samples) for each coral species, as well as the microbes that are consistently present at each location (locally stable microbial associates, LSMA). Since many of the OTUs that were identified as part of the core microbiome, an LSMA and/or differentially abundant were also present in the seawater, we used *DESeq2* to assess which OTUs were more abundant in the seawater than the bacterial communities of the coral to identify potential transiently associated microbes, e.g., environmental microbes trapped in the coral mucus.

The PICRUSt2 (Phylogenetic Investigation of Communities by Reconstruction of Unobserved States) pipeline (Langille et al., 2013; Douglas et al., 2020) was used to identify OTUs putatively involved in the nitrogen and sulfur cycles. Based on the Kyoto Encyclopedia of Genes and Genomes (KEGG^2^) (Kanehisa and Goto, 2000), the relevant KEGG orthologs were selected (Supplementary File S7-1). OTUs that had a Nearest Sequenced Taxon Index (NSTI) value of <2 and that were predicted by PICRUSt2 to contain at least one KEGG ortholog involved in the each step of a nutrient cycling process (Nitrogen Cycle: nitrogen fixation, nitrification/ammonium oxidation, nitrate reduction/ammonification and/or denitrification, Sulfur Cycle: DMSP demethylation or cleavage, sulfur oxidation and/or sulfate reduction) were selected. The nitrite-to-nitrate step in the nitrification process is, however, only performed by highly specialized microbes possessing the *nxrAB* genes, but KEGG orthologs K00370 and K00371 also contain the common *narGZHY* genes involved in denitrification and dissimilatory nitrate reduction. Therefore, we ensured that only taxa known to be capable of performing this step (e.g., *Nitrospina*, *Nitrospira*) were included. Differences in the relative abundances between locations and time points of functional groups overall were analyzed using a negative binomial generalized linear model using the R-package *MASS* (Venables and Ripley, 2002) followed by pairwise comparisons with the R-package *multcomp* (Hothorn et al., 2008).

## Results

### Microbial Community Diversity

**FIGURE 3:**
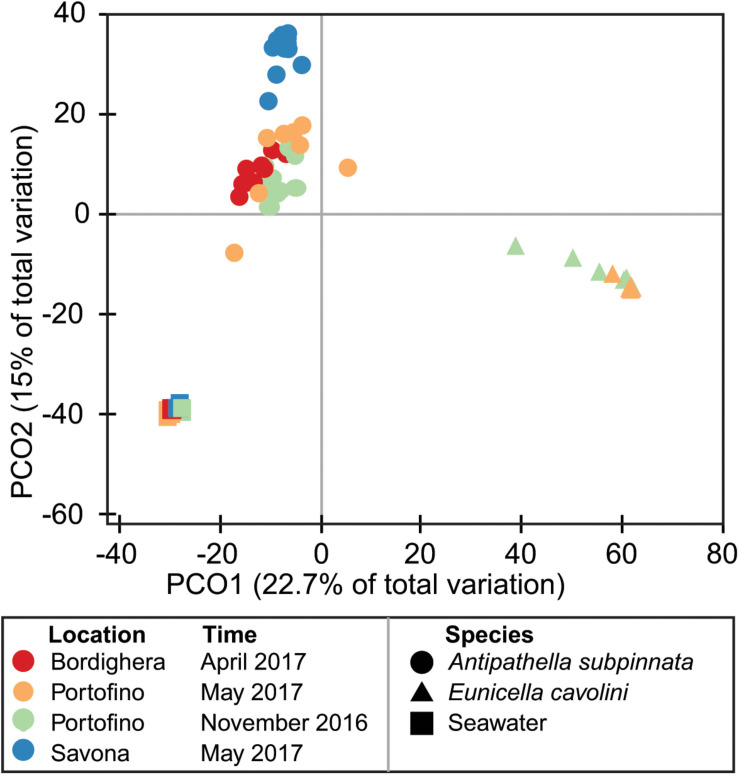
Differences in the diversity of the *Antipathella subpinnata* and *Eunicella cavolini* microbiomes. Beta diversity of the microbiota of *A. subpinnata* and *E. cavolini* and the surrounding seawater is presented in a principal coordinates analysis (PCoA) ordination plot based on a Bray–Curtis similarity distance matrix.

Beta diversity analyses (Supplementary File S2: Supplementary Table S1) showed that the prokaryotic communities of both the black coral *Antipathella subpinnata* and the gorgonian *Eunicella cavolini* were distinct from the surrounding seawater at each location and time point (all comparisons *p* ≤ 0.0036, Figure 3). However, these analyses also indicated that the prokaryotic community of *A. subpinnata* was different (I) between the three sampling locations (all comparisons *p* < 0.0003, Supplementary Figure S1A) as well as (II) between autumn and spring at the Portofino location (*p* < 0.0005, Figures 1A,B) and (III) compared with the sympatric *E. cavolini* at both time points (both comparisons *p* ≤ 0.0002, Figure 3). In addition, the microbial communities of the seawater also showed spatial and temporal differences (spatial *p* ≤ 0.023, temporal *p* < 0.0068, Supplementary Figure S1C). Contrastingly, no temporal differences were observed in the microbial community associated with *E. cavolini* (*p* = 0.3079, Figure 3).

Outcomes of the analyses of the three alpha diversity metrics richness (observed OTUs), evenness (Simpson’s *E*), and diversity (Shannon-Wiener *H*) are provided in Supplementary File S2: Supplementary Table S2–S3. In the spring of 2017, no significant differences in evenness and diversity were observed between *A. subpinnata* and seawater, except at the Savona shoal (*p* = 0.0001). In addition, the prokaryotic communities of both *A. subpinnata* and the seawater showed significant spatial differences (*p* < 0.02), however, no difference was found in the microbiota of *A. subpinnata* collected near Portofino and Bordighera. At the Portofino location, significant differences in the richness of the microbiota were found between the coral species and seawater. A similar pattern was observed in microbiota diversity, with the exception that the diversity in the seawater and in the *A. subpinnata*-associated microbiota was similar. No temporal differences were observed in any of the alpha diversity metrics.

### Prokaryotic Community Composition and ‘Core Microbiome’

Overall, we observed 7813 different bacterial and 73 archaeal OTUs associated with *A. subpinnata*. In contrast, 343 bacterial OTUs and no archaeal OTUs were found in the microbiota of *E. cavolini*. Archaea were relatively low abundant in the microbiota of *A. subpinnata* in comparison with bacteria, representing only 0.25% of the prokaryotic community associated with this black coral near Portofino, 0.17% near Bordighera and 0.02% near Savona.

**FIGURE 4:**
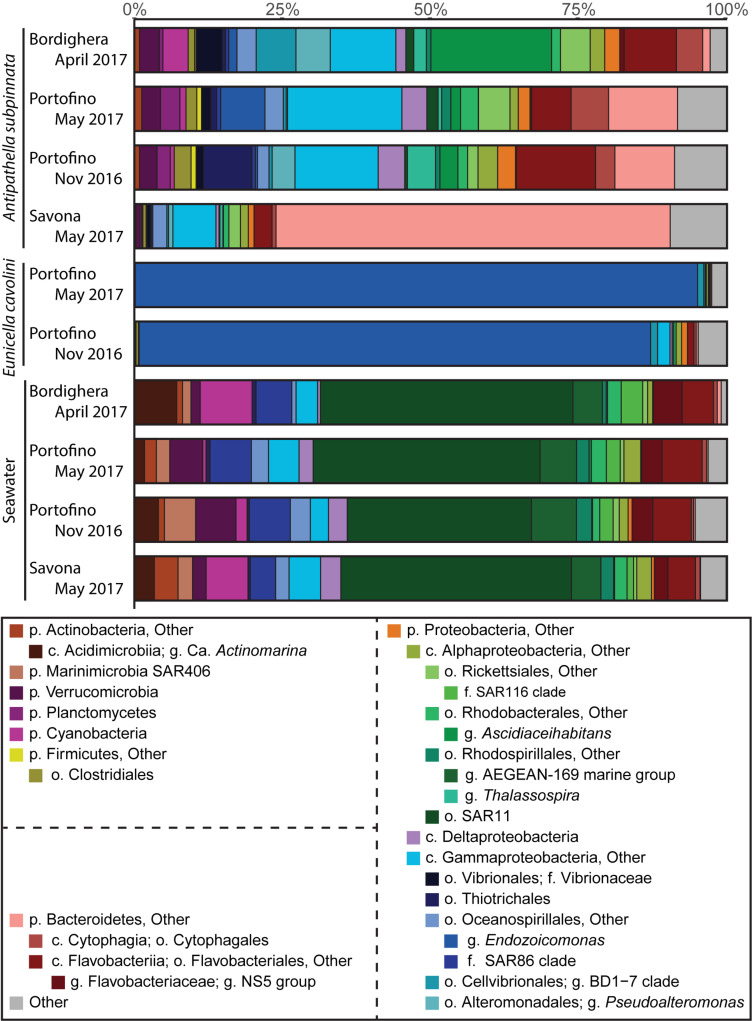
Overview of the composition of the bacterial community associated with the black coral *Antipathella subpinnata* and in the seawater at three locations (Bordighera, Portofino and Savona), and at two time points (spring and autumn) at the Portofino location. The composition of the microbiota of the gorgonian *Eunicella cavolini*, living sympatrically with *A. subpinnata* at Portofino, is also provided. The composition of the microbial communities is presented at the various taxonomic levels (p, phylum; c, class; o, order; f, family; g, genus). The contribution of each taxon is indicated in percentages (%). Taxa with an abundance of <1% were merged into its corresponding higher taxonomic level. Higher level taxa indicated with ‘Other’ (e.g., p. Proteobacteria, Other) include all subtaxa not specified below. Other (gray) contains all phyla with an abundance of <1%.

**FIGURE 5:**
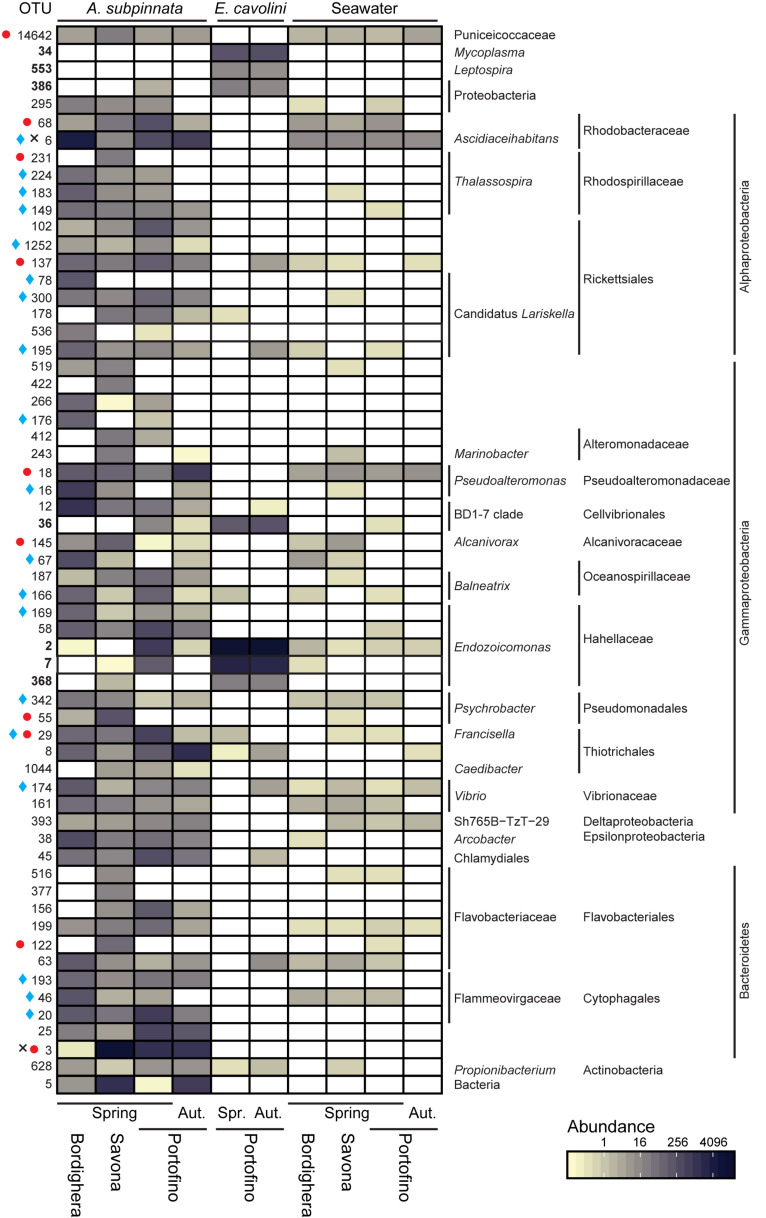
Core and locally stable microbial associates of *Antipathella subpinnata* and *Eunicella cavolini* and their presence in the surrounding seawater. Heatmap presents the abundance of OTUs considered as part of the core microbiome (present in all samples of a species) and locally stable microbial associates (LSMA, present in at least 80% of the samples from one location). OTUs indicated in bold are core microbes of *E. cavolini.* LSMA OTUs indicated with a symbol are present in all samples of *A. subpinnata* at a given location: Bordighera (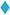), Savona (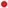) and Portofino (**×)** in spring (Spr., April/May 2017) and autumn (Aut., November 2016). The taxonomy of each OTU is given at lowest possible taxonomic level. OTUs presented have a higher relative abundance in *A. subpinnata* than seawater. See Supplementary Figure S3 for OTUs with equal or lower relative abundance in *A. subpinnata* than seawater.

Clear differences in the prokaryotic community composition between *A. subpinnata* and *E. cavolini* were indeed observed (Figure 4). The bacterial community of *A. subpinnata* was composed of a range of Proteobacteria (particularly Gamma-, Alpha- and Deltaproteobacteria), Bacteroidetes, Firmicutes, Cyanobacteria, Planctomycetes and Verrucomicrobia (Figure 4). Differences in the relative abundances of these taxa existed between locations and time points (Figure 4, discussed below), but also among individual colonies within the different locations (Supplementary Figure S2). No ‘core microbiome’ (i.e., those microbes that are ubiquitous and consistently present within the microbiota of a species, regardless of space and time) could be determined for *A. subpinnata*. However, we identified 94 OTUs that were present in at least 80% of the samples at one of the locations. Twenty-one of those OTUs were significantly more abundant in seawater and belonged to known bacterioplankton taxa (e.g., SAR11, SAR116, AEGEAN-169 and Flavobacteriaceae NS marine groups, Supplementary Figure S3) and were therefore considered transient associates. Of the remaining 73 locally stable microbial associates (LSMA) of *A. subpinnata* 54 OTUs were significantly more abundant in *A. subpinnata* than seawater (Figure 5), while for 19 OTUs no difference was found (Supplementary Figure S3). The relative abundance of these LSMAs within the bacterial communities of *A. subpinnata* differed between on average 29 and 82% depending on the location and time point (Supplementary File S3). LSMAs belonged primarily to the main taxa identified (relative abundance > 1%, Figure 4 and Supplementary File S4), particularly Rhodobacteraceae, *Thalassospira*, Rickettsiales, *Pseudoalteromonas*, Cellvibrionales BD1-7, *Vibrio*, Thiotrichales and various taxa within the phylum Bacteroidetes. An archaeon of the order Thermoplasmatales (OTU1066) was also found commonly associated with *A. subpinnata* (relative abundance ∼0.05%) from the Bordighera (9/10 samples) and Portofino (9/10 and 5/10 samples in November and May, respectively) populations, but it was absent in the Savona population (Supplementary Figure S3).

The bacterial communities associated with *E. cavolini* were dominated by bacteria from the genus *Endozoicomonas* and to a lesser extent the Cellvibrionales Clade BD1-7 (Figure 4). OTUs belonging to these taxa were the major constituents of the *E. cavolini* core microbiome, which consisted of 7 OTUs that represented 89–97% of the associated bacterial community. OTUs identified as *Leptospira*, *Mycoplasma* and an unidentified Proteobacterium were also part of the core microbiome of this gorgonian (Figure 5 and Supplementary Files S2, S3).

### Spatial Differences in the Black Coral-Associated Bacterial Communities

**FIGURE 6:**
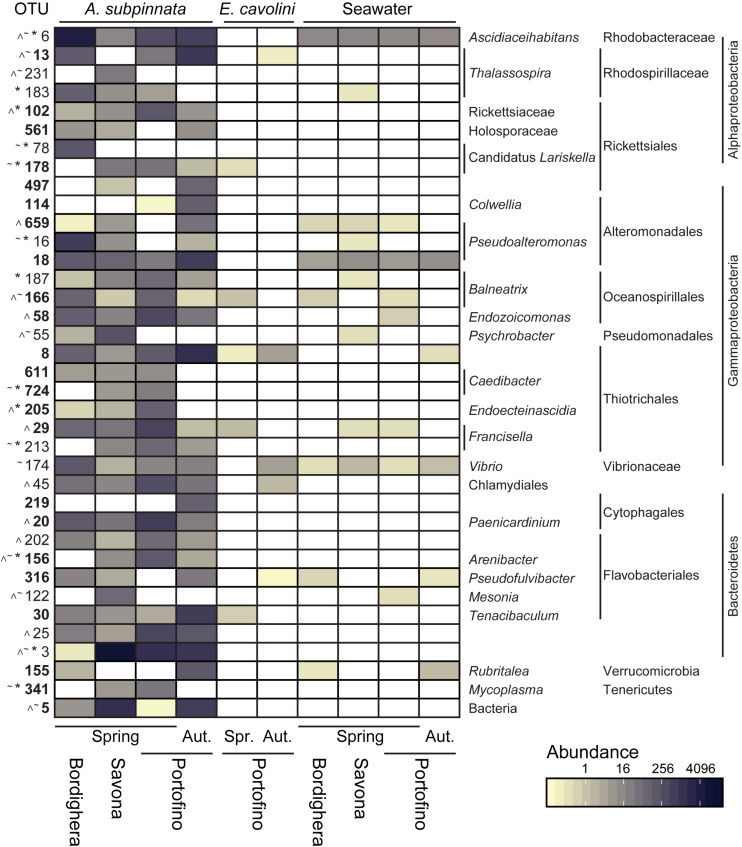
Microbes associated with *Antipathella subpinnata* that show differential abundance between locations or time points. Heatmap presents the abundance of OTUs found differentially abundant between locations (Bordighera, Savona and Portofino) in the spring (Spr., April/May 2017) and/or between spring and autumn (Aut., November 2016) at the Portofino location. Abundance of these OTUs in the microbiota of *E. cavolini* and seawater is also provided. The taxonomy of each OTU is given at the lowest possible taxonomic level. Symbols indicate OTUs showing spatial differences (∼ Bordighera versus Savona, * Bordighera versus Portofino, ^ Savona versus Portofino). OTUs in **bold** were found differentially abundant between time points. OTUs presented have a higher relative abundance in *A. subpinnata* than seawater. See Supplementary Figure S4 for OTUs with equal or lower relative abundance in *A. subpinnata* than seawater.

Using differential abundance analysis (Supplementary Files S5A–C), 101 OTUs were found to be primarily responsible for the spatial differences in the prokaryotic communities of *A. subpinnata*. However, 9 of these OTUs were more abundant in the surrounding seawater and were therefore likely environmental microbes trapped in the coral mucus (Supplementary Figure S4). Nearly half of the remaining OTUs matched the LSMA criterion (22 OTUs were present in at least 80% of samples at one location and 19 OTUs were present in at least 100% of the samples at one location) (Figure 6 and Supplementary Figure S3). The spatial differences observed in the diversity of the bacterial communities of *A. subpinnata* in May 2017 (Supplementary Figure S1A) could be discerned in the community composition (Figure 4) and attributed primarily to changes in the abundances of OTUs belonging to the main phyla (Figure 6 and Supplementary Figure S4).

The microbial communities associated with *A. subpinnata* at Savona were highly distinct from those at Portofino and Bordighera (Figure 4). This was primarily driven by the high relative abundance of Bacteroidetes LSMA OTU3 and the unknown Bacteria OTU5, which represented respectively ∼66 and 7.5% of the bacterial communities at Savona. Contrastingly, these OTUs were significantly lower or nearly absent in the Bordighera (0.0006 and 0.05%) and Portofino (7.1% and 0%) populations (Figure 6 and Supplementary File S3). Consequently, the relative abundances of nearly all other taxa were lower in comparison with Portofino and Bordighera (Figure 4). The exceptions identified included several Flavobacteriales OTUs that were only present in Savona and higher abundances of the Gammaproteobacteria *Psychrobacter* OTU55 and Alteromonadaceae (Figure 6 and Supplementary Figure S4). We also observed significantly higher numbers of the hydrocarbon-degrading gammaproteobacterial genera *Alcanivorax*, *Oleiphilus*, and *Marinobacter* in the Savona population (Supplementary Figure S4). These genera represented 1.90% of the bacterial community compared with 0.03 and 0.2% in Portofino and Bordighera, respectively.

The *A. subpinnata*-associated prokaryotic communities at Bordighera and Portofino in May 2017 were more similar to each other than at Savona (Supplementary Figure S1A and Figure 4), but compositional differences were still observed. Most clearly visible were the significantly higher abundances of *Ascidiaceihabitans*, *Pseudoalteromonas*, *Colwellia*, Vibrionaceae and numerous Bacteroidetes phylotypes at Bordighera (Figures 4, 6 and Supplementary Figure S4). On the other hand, the Portofino *A. subpinnata* colonies harboured significantly higher levels of Planctomycetes, Thiotrichales, Bacteroidetes (OTU25 and Cytophagales OTU20), Chlamydiales OTU45, Rickettsiaceae OTU102, *Mycoplasma* and *Endozoicomonas* (Figure 6 and Supplementary Figure S4). Two of the three *Endozoicomonas* OTUs (OTU2 and 7) were the main core bacterial symbionts of the sympatric gorgonian *E. cavolini* (Figure 5 and Supplementary Figure S4) and were exclusively found in *A. subpinnata* at Portofino.

In addition, we found differences in the OTUs belonging to specific genera present on *A. subpinnata* at the different sampling locations. In the case of the Rhodospirillales genus *Thalassospira*, for example, OTU13 was present in the Bordighera and Portofino populations but absent from the Savona population, whereas OTU231 was dominant in *A. subpinnata* near Savona but absent at the other locations (Figure 6). Similarly, OTU78 was a Rickettsiales *Ca. Lariskella* phylotype only found in Bordighera, while OTU178 was present only in Savona and Portofino (Figure 6). A third example concerned the genus *Rubritalea* of the Verrucomicrobia: OTU155 and OTU361 were observed in the microbiota of *A. subpinnata* near Bordighera, but OTU53 and OTU695 were detected in the Portofino and Savona populations (Figure 6). Although these patterns were obvious, the relevance of these spatial differences within particular genera for holobiont functioning and acclimation remains unclear.

### Temporal Differences in the Black Coral-Associated Bacterial Communities

At the Portofino location, we also observed temporal changes in the diversity of the microbial community associated with *A. subpinnata* (Supplementary Figure S1B). Samples collected in November 2016 contained higher levels of Flavobacteriales, Rhodospirillales (genus *Thalassospira*), Planctomycetes, Alteromonadales and Thiotrichales compared with May 2017, but lower levels of *Endozoicomonas* and Rickettsiales bacteria (Figure 4). These results were confirmed by differential abundance analysis, identifying 129 OTUs (incl. 18 that likely belong to the bacterioplankton) that were differentially abundant between time points (Figure 6, Supplementary Figure S4, and Supplementary File S5D). Of these OTUs, 65 were only present in the *A. subpinnata* microbiota in November and 23 OTUs were exclusive to May, explaining the significant differences observed in the diversity of these bacterial communities. It should also be noted that we cannot exclude the possibility that the microbiota of individual colonies are stable over time but differ between neighboring colonies (Supplementary Figure S2), as we did not sample the same colonies at both time points. In contrast, no OTUs were found differentially abundant in *E. cavolini* between those two time points (Supplementary File S5E).

### No Relationship Between Differences in the Bacterioplankton and Microbiota of *A. subpinnata*

As significant spatial and temporal differences were also observed in the seawater, we hypothesized that seawater microbes trapped in the *A. subpinnata* mucus may be primarily responsible for the differences observed. However, we found that only a small portion of the OTUs differentially abundant in the coral microbiota (spatial: 7–18%, temporal 25%) were also differentially abundant in the seawater (Supplementary File S6). In addition, we found that some of these overlapping OTUs had contrasting abundance patterns. For example, OTU33 and OTU41 were significantly lower in the *A. subpinnata*-associated bacterial communities in Portofino than in Savona in May 2017, however, their abundance in the seawater was higher in Portofino than in Savona (Supplementary Figure S3). Besides, several OTUs that were differentially abundant in both the seawater and coral microbiota had an overall higher abundance in corals than seawater (Supplementary Figure S3), suggesting that these may thus be coral symbionts rather than trapped environmental microbes.

### Predicted Microbiome Functionality: Nutrient Cycles in the *A. subpinnata* Holobiont

**FIGURE 7:**
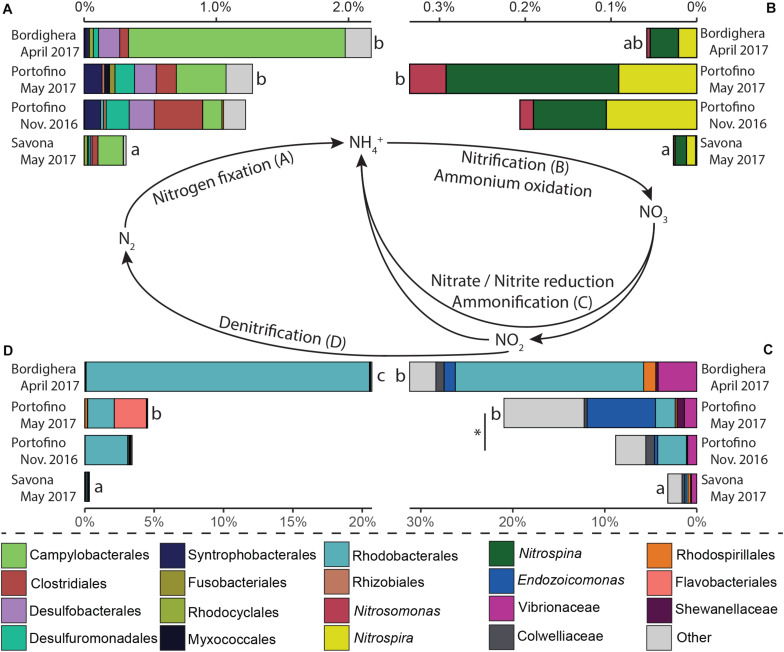
Relative abundance of *Antipathella subpinnata-*associated bacteria with a putative role in the nitrogen cycle. The nitrogen cycle consists of four main processes and microbes putatively involved in these processes are presented: **(A)** nitrogen fixation, **(B)** nitrification/ammonium oxidation, **(C)** nitrate/nitrite reduction/ammonification and **(D)** denitrification. Differences between locations are indicated with letters, differences between autumn and spring in the Portofino population are indicated with an asterisk (*). Taxa with low abundances are combined and indicated in light gray.

**FIGURE 8:**
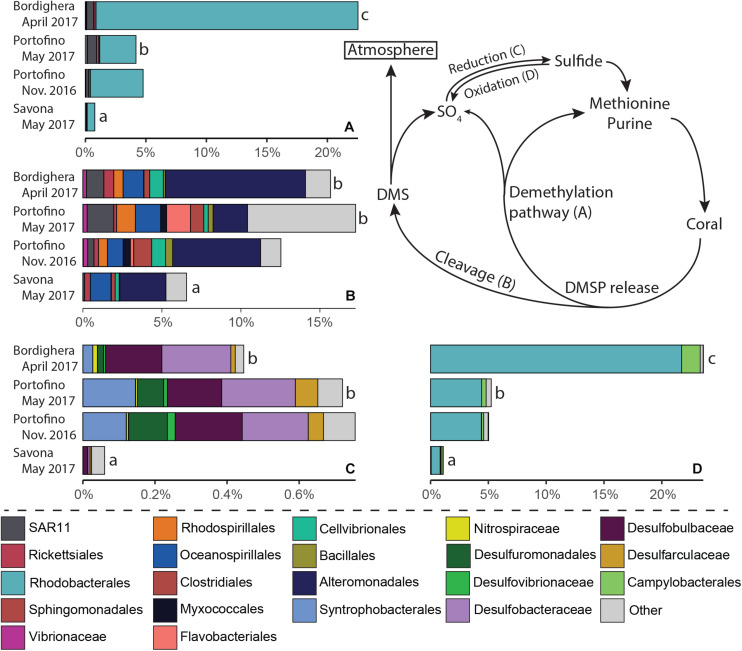
Relative abundance of *Antipathella subpinnata-*associated bacteria with a putative role in the sulfur cycle. The sulfur cycle consists of four main processes and microbes putatively involved in these processes are presented: **(A)** DMSP demethylation, **(B)** DMSP cleavage, **(C)** sulfate reduction and **(D)** sulfur oxidation. Differences in the abundance of functional groups between locations are indicated with letters. Taxa with low abundances are combined and indicated in light gray.

PICRUSt2 analysis identified a number of taxa involved in one or multiple steps of the nitrogen (Figure 7, nitrogen fixation, nitrification/ammonium oxidation, nitrate reduction/ammonification and denitrification) and/or sulfur (Figure 8, DMSP demethylation or cleavage, sulfur oxidation, sulfate reduction) cycles (Supplementary File S7). Microbes likely involved in the different steps of these two nutrient cycles were present in the *A. subpinnata* holobiont at each location and at both time points at the Portofino site (Figures 7, 8). However, significant temporal and spatial differences in the relative abundances of the assigned functional groups and their members were observed (statistical outcomes: Supplementary File S8). The relative abundances of all functional groups were significantly lower in the Savona population compared with the other locations (Figures 7, 8, all comparisons *p* < 0.02, Supplementary File S7). This was likely related to the high relative abundance of Bacteroidetes at this location (Figure 4), resulting in generally low relative abundances of all other taxa, including those involved in the nitrogen and sulfur cycles. Differences in functional groups were also observed between the Bordighera and Portofino populations, with *A. subpinnata* near Bordighera harboring higher levels of potential denitrifying (Figure 7D, *p* = 0.0001), DMSP-demethylating (*p* = 1^∗^10–9, Figure 8A) and sulfide-oxidizing (*p* = 1^∗^10–10, Figure 8D) bacteria, especially Rhodobacterales. Temporal differences could only be discerned in the relative abundances of nitrite- and nitrate-reducing bacteria (*p* = 0.0008), which may have been particularly related to the higher relative abundances of *Endozoicomonas* in May compared with November (Figure 7C).

## Discussion

We present one of the first in-depth assessments of the bacterial communities associated with black corals, which provide crucial forest-like structural habitat in the mesophotic and deep sea, by profiling its associated microbial community on both spatial and temporal scales. Our findings show that *Antipathella subpinnata* possesses a microbiome distinct from the surrounding seawater as well as from a sympatric arborescent gorgonian octocoral. The potential lack of a true core microbiome and the significant differences in bacterial communities between locations and sampling time points suggest that the composition of this holobiont is highly influenced by local environmental conditions. Here, we discuss (1) the composition of the black coral microbiota in the context of coral microbial ecology, (2) the putative functions of the prokaryotes associated with *A. subpinnata*, (3) the potential causes of the high variability in the microbiota of this black coral, and (4) the implications that the lack of a ‘core microbiome’ may have for this coral species as well as for the field of coral microbial ecology in general.

### Microbiome of *Antipathella subpinnata*

Our study shows that the microbiome of the black coral *A. subpinnata* was dominated by Proteobacteria (particularly Gamma-, Alpha-, and Deltaproteobacteria), Bacteroidetes and to a lesser extent by Firmicutes, Cyanobacteria, Planctomycetes, and Verrucomicrobia. When placing these results in context with the five other studies on black coral-associated microbes published to date (Penn et al., 2006; Santiago-Vázquez et al., 2007; Zhang et al., 2012; Dannenberg, 2016; Liu et al., 2018), some interesting patterns emerge: namely, the bacterial communities (classified at the genus level and higher) may be relatively conserved across black coral species. For example, *Leiopathes glaberrima*, a deep-sea species with a wide distribution (Caribbean, Pacific, and Mediterranean), possesses an associated microbial community that is composed mostly of Proteobacteria, Bacteroidetes, Firmicutes, and Actinobacteria (Dannenberg, 2016). In addition, initial culture-based techniques (Santiago-Vázquez et al., 2007; Zhang et al., 2012) isolated and identified various bacteria from black corals that belong to taxa which are now found widespread in the microbiota of black corals based on culture-independent techniques (here in *A. subpinnata* and Dannenberg, 2016; Liu et al., 2018). These taxa include Firmicutes (e.g., *Bacillus*), Actinobacteria (e.g., *Propionibacterium*) and Gammaproteobacteria (e.g., *Acinetobacter*, *Pseudomonas*, *Pseudoalteromonas*, *Psychrobacter*, *Vibrio*). This also suggests that many of the bacterial symbionts of black corals may be amenable to cultivation, allowing more detailed studies of their function.

The bacterial communities of *A. subpinnata* and other black corals were also found to share significantly more characteristics of the microbial communities associated with other Hexacorallia than with Octocorallia. The holobiont of Mediterranean *A. subpinnata* showed similarities with the Mediterranean corals *Oculina patagonica* and *Cladocora ceaspitosa*. These shallow scleractinian corals also harbor relatively high levels of Bacteroidetes and Alpha-, Delta- and Gammaproteobacteria (∼60% of the overall community) and lower levels of Verrucomicrobia, Planctomycetes, Cyanobacteria and Firmicutes (Rubio-Portillo et al., 2018b; Bednarz et al., 2019). Although at relatively lower abundances, bacteria from these taxa are also commonly found in sea anemones (Brown et al., 2017; Herrera et al., 2017), tropical scleractinian corals (Sunagawa et al., 2010; Godwin et al., 2012; Morrow et al., 2012) and numerous deep-sea coral species (Kellogg et al., 2009, 2016, 2017; Gray et al., 2011; Lawler et al., 2016; Kellogg, 2019). Contrastingly, the *A. subpinnata* prokaryotic community composition did not show much similarity with the bacterial communities of the gorgonian *Eunicella cavolini*, despite living sympatrically in the same location, and having a similar arborescent colony structure and ecological function.

In conclusion, we show that, despite the lack of a ‘core microbiome’ at the OTU level, the *A. subpinnata*-associated prokaryotic community is primarily composed of a limited number of bacterial taxa. Most of these higher level taxa have also been found in the microbial community of other black coral species. These similarities suggest that black corals may have a relatively conserved bacterial community at the taxonomic genus level and up.

### Putative Functions of *Antipathella subpinnata*-Associated Prokaryotes

Although many studies have addressed the composition of the coral-associated microbial community under natural as well as experimental conditions, still very little is known about the functions of these bacteria. As such, it is difficult to understand the role of the microbes within the *A. subpinnata* holobiont. Based on functional and genomic studies on bacteria with closely related 16S rRNA gene sequences, it might be possible to infer the role of coral-associated microbes, which may provide some insights into their niche within the holobiont. However, it is important to remain cautious when inferring microbial functions, as lateral gene transfer among bacteria and mutations may have altered a microbe’s catabolic and anabolic capacities and behavior, compared with its taxonomically close relatives.

The majority of the dominant bacterial taxa found in the prokaryotic communities of *A. subpinnata* belong to taxa which are commonly found within the holobiont of benthic marine invertebrates, including corals. Some of these microbes may have a role in nutrient cycling, which would be of high importance to the health and nutritional status of the coral holobiont. For example, bacteria in the phylum Bacteroidetes play an important role in organic carbon cycling in the marine environment (Thomas et al., 2011; Fernández-Gómez et al., 2013). They are particularly recognized for their capacity to break down complex organic molecules, including chitin. As such, these bacteria may aid the coral host with the digestion of captured prey. This may also be the function of the relatives of *Vibrio gigantis* that we found in *A. subpinnata*, as *V. gigantis* is a mutualist in shellfish (Le Roux et al., 2005) and sea cucumbers (Beleneva and Kukhlevskii, 2010) that aids in the host’s food digestion using a broad spectrum of enzymes, including chitinases. However, as the skeletons of black corals have generally a high (∼10–15%) chitin content (Goldberg et al., 1994; Bo et al., 2012a), these bacteria may potentially also use the chitin produced by the coral as a carbon and nitrogen source.

One of the best studied coral-associated bacterial taxa, *Endozoicomonas*, was also commonly present in the *A. subpinnata* and *L. glaberrima* (Dannenberg, 2016) holobionts, although at relatively low abundances. Contrastingly, it is the main symbiont of *E. cavolini* (Bayer et al., 2013; van de Water et al., 2017, 2018b). Genome analyses have indicated that *Endozoicomonas* may provide its host with a variety of amino acids, which are synthesized using ammonium and sulfur acquired through its involvement in nutrient cycling processes of nitrogen (nitrate reduction and ammonification) and sulfur (DMSP metabolism) (Neave et al., 2016). This led us to investigate whether other bacteria involved in the (re)cycling of essential nutrients (nitrogen, sulfur, and phosphorus) were present in the microbiota of *A. subpinnata*. Overall, we found bacteria putatively involved in all steps of the nitrogen cycle in the black coral-associated microbial community (Figure 7). The presence of these bacteria may indicate that the *A. subpinnata*-associated bacterial communities are able to acquire (via nitrogen fixation) and retain (via nitrification and ammonification) nitrogen within the holobiont. Nitrogen fixation has been shown in the deep-sea coral *L. pertusa* (Middelburg et al., 2015) and a complete nitrogen cycle may be present in the microbiota of several deep-sea octocoral species (Kellogg et al., 2016; Lawler et al., 2016). However, the relatively high levels of potential denitrifying bacteria in comparison with nitrogen-fixing bacteria in the microbiota of *A. subpinnata* could indicate that there might be a net loss of nitrogen, requiring the holobiont to obtain nitrogen from exogenous sources, such as predation on plankton (Coppari et al., 2020).

Phosphorus is generally a limiting nutrient for organismal growth, particularly in the marine environment and efficient (re)cycling is therefore of high importance for a holobiont. In addition to the uptake of inorganic phosphate, phosphonates are another main source of phosphorus in the marine environment. Although primarily a microbial process, some marine invertebrates (incl. corals and anemones) possess genes representing the complete pathway for phosphonate synthesis (Shoguchi et al., 2013) and contain high levels in their tissues (Henderson et al., 1972; Garrett et al., 2013; Godinot et al., 2016). In two soft corals, microbes that are able to degrade the highly stable C-P bonds of phosphonates have previously been found, including *Thalassospira*, *Vibrio*, *Pseudoalteromonas*, *Psychrobacter* and Bacteroidetes (Thomas et al., 2009). These taxa were also commonly found in the microbiota of *A. subpinnata*, indicating that phosphorus cycling likely exists within the black coral holobiont, thereby limiting the loss and facilitating the acquisition of this crucial nutrient.

We also found several microbial taxa linked to the degradation of dimethylsulfoniopropionate (DMSP), a sulfur compound shown to be produced at high levels by coral animals (Raina et al., 2013). The sulfur contained in DMSP can be recycled by a large number of bacterial taxa using the demethylation pathway (Figure 8), and is then used in the synthesis of purines and the essential amino acid methionine. The other DMSP degradation pathway leads to the cleavage of DMSP into DMS by bacteria (Figure 8). Although sulfur may be lost after oxidation by bacteria in the form of sulfate in both pathways, sulphite- and sulfate-reducing bacteria (Figure 8) can reduce this and environmental sulfate into bioavailable sulfide for incorporation into biomolecules. The common presence of DMSP-metabolizing and sulfate-reducing bacteria in the microbiota of *A. subpinnata* shows that sulfur cycling likely takes place within the black coral holobiont. Although the metabolic activities of these microbes are unknown, the relatively high levels of sulfide-oxidizing bacteria in comparison with sulfate-reducing bacteria may indicate a net loss of sulfur.

DMSP has also been implicated in the structuring of the microbiota of corals (Frade et al., 2016), and may be used by coral-associated microbes to produce antimicrobial compounds capable of eliminating coral pathogens (Raina et al., 2016). This shows the importance of some bacteria in microbial community regulation of the coral holobiont. This might also be important in *A. subpinnata* as *Vibrio* bacteria were often present at low abundance in its microbiota. These bacteria are commonly found in corals, particularly in the surface mucus layers (Bourne and Munn, 2005; Chimetto et al., 2009; Raina et al., 2009; Porporato et al., 2013; Ainsworth et al., 2015), where they are considered to be commensal or opportunistic pathogens because of their implication in various coral diseases (Ben-Haim et al., 2003; Ushijima et al., 2012; Munn, 2015; Rubio-Portillo et al., 2018a). Some *A. subpinnata*-associated bacteria, for example *Pseudoalteromonas*, may have a microbiome regulatory role. These bacteria are often found in association with marine animals, including cnidarians, and are known to secrete compounds with antibacterial (Holmström and Kjelleberg, 1999; Shnit-Orland et al., 2012; Richards et al., 2017), antifungal (Shiroyama et al., 2017) and alginolytic (Holmström and Kjelleberg, 1999) activities. Besides, *Endozoicomonas* (Neave et al., 2016) and Actinobacteria (phylum including *Propionibacterium*) (Ritchie, 2006; Nithyanand et al., 2011; Krediet et al., 2013; Zhang et al., 2013) have been implicated in coral microbiome regulation.

Overall, it appears that each step in the cycling of nutrients may be performed by a number of different bacterial taxa present in the *A. subpinnata* holobiont. This suggests that functional redundancy may be present within the *A. subpinnata* microbiota. However, it also implies that, rather than having a true ‘core microbiome,’ this black coral holobiont may ensure the presence of important core functions within its microbiome, with a potential microbiome regulatory role for some of its common microbial associates.

### Potential Causes of Variability in the *Antipathella subpinnata* Microbiota

The microbial communities associated with *A. subpinnata* were found to be distinct at each of the three locations and different between the spring and autumn. As all colonies were visually healthy at the time of collection, a cause for these differences is difficult to identify. However, it was unlikely to be related to the trapping of bacterioplankton in the large amounts of mucus produced by this black coral (Bo et al., 2008), because (1) only a minor fraction of the differentially abundant OTUs in the coral-associated bacterial communities was also differentially abundant in the seawater, and (2) the relative abundance of some OTUs was higher in the coral than seawater and these may thus have been released through shedding – a known mechanism of corals to regulate their microbiome (Garren and Azam, 2012).

The genetic structure of the black coral populations, and thus host genotype, may be a driver of the spatial differences observed in the prokaryotic communities associated with *A. subpinnata*. Possible links between host genotype and microbiome were recently found in two deep-sea octocoral species from the genus *Primnoa* (Goldsmith et al., 2018) and spatial differences in the microbiota were also observed in two Caribbean populations of *L. glaberrima* (Dannenberg, 2016). However, preliminary analyses have revealed that coastal *A. subpinnata* populations on the western coast of Italy are highly connected (Costantini et al., 2019). This suggests that genotype has a limited role, and that the *A. subpinnata*-associated microbial communities were likely influenced by local environmental conditions.

Increased seawater temperature (Bourne et al., 2007; Ziegler et al., 2017; Pootakham et al., 2018) and anthropogenic stressors (van de Water et al., 2017, 2018b), including pollution (Ziegler et al., 2015; Leite et al., 2018), have previously been linked to shifts in the coral microbiome. But in our study, the seawater temperatures at the time of sampling were nearly the same at all locations and the shoals are not exposed to heavy river run-off pollution due to their relative distance from shore and depth. We did, however, observe a significantly higher abundance of bacteria that are capable of degrading or exclusively feeding on hydrocarbons in the microbiota of *A. subpinnata* of the Savona population, such as *Marinobacter*, *Alcanivorax*, and *Oleiphilus*. This may be linked to nearby activities, as the Port of Savona (incl. Vado Ligure) handles petroleum products, harbors a large fleet of commercial fishing vessels and is frequented by cruise and cargo ships, whereas the ports of the other two localities are primarily used for yachting. It is tempting to speculate that these microbes may convert these hydrocarbons into a bioavailable carbon source for the coral host as has been previously hypothesized for deep-sea corals from the Red Sea (Röthig et al., 2017). While this may partially explain the highly different *A. subpinnata*-associated bacterial communities near Savona, this does not account for all spatial and temporal differences observed.

The local conditions at each of our sampling sites were likely somewhat different in terms of silting levels, hydrodynamism and upwellings due to nearby canyons. In addition, modeling of the coastal currents in the Ligurian coastal region has shown that coastal mesoscale eddies are common and long-lived (over a month) (Casella et al., 2011). Particularly, the anti-cyclonic eddies between the coast and the main Ligurian-Provencal-Catalan current induce strong upwelling on their coastal side and are an important source of nutrients for ecosystems in the upper sea layer (down to 200 m depth) (Casella et al., 2011). Unfortunately, we were unable to verify whether such eddies were present at the time of sampling at any of the locations or how much time had passed since the last upwelling, and no data on nutrient levels at these locations are available. Regardless, our best explanation of our results is that the observed differences in the bacterial communities of *A. subpinnata* are due to the different local environmental conditions that characterize the sites and with a potential role of anti-cyclonic vortices.

Environmental conditions may also impact the main food source of the filter-feeding *A. subpinnata*: plankton. It was demonstrated recently that *A. subpinnata* in the Portofino area feeds primarily on mesozooplankton in spring, but nano- and picoplankton (incl. the bacterioplankton) in autumn (Coppari et al., 2020), which coincides with the temporal differences observed in the microbiota. Changes in food sources are known to affect the nutritional status and the microbiomes of organisms. Although a definitive link has not been established here, it is tempting to speculate that the diet of this black coral influences its microbiome and thereby potentially its function. The observed differences in the abundance of microbes involved in various steps of the nitrogen and sulfur cycles may reflect changes in the coral’s nutritional status and fitness. However, it could also be indicative of a change in the nutritional needs of the holobiont and an adjustment of the microbiome to fulfill these needs to improve host fitness. To address this, further studies linking coral physiology with the activity of host-associated bacteria will be required along with investigations into the black coral holobiont’s capacity to actively regulate its microbiome.

### Microbiome Flexibility in Black Corals and the Absence of a Bacterial ‘Core Microbiome’

Black corals are one of the longest lived animals known, with a reported colony age of up to 4,265 years for *L. glaberrima* (Roark et al., 2009). As they are sessile animals, it is imperative for their survival to employ strategies that allow them to acclimate rapidly in response to environmental change. Actively changing the microbiota may represent one such mechanism. Given the significant differences in microbiota observed on spatial and temporal scales in healthy populations of *A. subpinnata*, our results indicate that the holobiont of this coral has indeed a high degree of microbiome flexibility. The degree of flexibility in holobiont structure and composition may, however, differ between species as demonstrated by Ziegler et al. (2019). They showed that the microbiota of the tropical reef-building hexacoral *Acropora hemprichii* responds to different levels of anthropogenic impacts and can even recover when transplanted to non-impacted sites. In contrast, they found that *Pocillopora verrucosa*’s microbiota remains stable regardless of the level of impact. Based on these differences in microbiome flexibility, the authors referred to corals that show microbial adaptation to the surrounding environment as “microbiome conformers,” and to corals that maintain a constant microbiome as “microbiome regulators” (Ziegler et al., 2019). While these terms provide guidance, most holobionts can likely not be strictly assigned to one group, and a spectrum of microbiome flexibility where the microbiome “regulators” and “conformers” are on either end of the scale should be considered. For example, *E. cavolini* possesses a microbiota dominated by its core microbiome, which shows little temporal differences but some spatial differences as seen here and previously (van de Water et al., 2017, 2018b). This suggests that this gorgonian is in the ‘microbiome regulator’ spectrum, as its microbiome is relatively stable but exhibits some variation, mostly in the abundances of core microbes. We also observed stability in the structural complexity of the microbiome of *E. cavolini* (based on alpha diversity metrics), which has been linked to tolerance of another coral in the ‘microbiome regulator’ spectrum to environmental change (Röthig et al., 2020). As all colonies of *A. subpinnata* sampled were visually healthy, the differences in the microbiota among populations and time points likely did not reflect an unhealthy state of the holobiont or a pathobiome. This black coral also did not completely alter its microbiota as numerous microbes were present at the three sampling locations. Instead, it is more likely that *A. subpinnata* is on the “microbiome conformer” side of the scale, adjusting its microbiota by selecting for the most beneficial microbial community depending on the environment (Reshef et al., 2006; Rosenberg et al., 2007) allowing it to cope with change. Consequently, the structural complexity of *A. subpinnata*’s microbiota was also significantly impacted, particularly near Savona.

However, it should be noted that 90–95% of the prokaryotic community of *A. subpinnata* was still composed of the main microbial taxa (those with an abundance > 1%) at all locations. This suggests that some degree of fidelity still exists within the composition of the holobiont, but not at the 97% phylotype/OTU level. Instead, black corals may rely on a ‘functional core’ (i.e., a complement of metabolic and other molecular functions that are performed by the microbiome but are not necessarily provided by the same organisms, Shafquat et al., 2014). This is in line with our results on the stable presence of microbes involved in nutrient cycling. Also Jaspers et al. (2019) determined that intimate associations between a host and its microbiota may not be a *sensu stricto* criterion for functional relevance as the composition of a holobiont may depend on multiple factors, including age, sex, life history and environment. Functional and/or metagenomics studies are, however, required to investigate whether changes in the microbiota affect the functioning of the coral holobiont.

Rigorous sampling at multiple locations, depths and over time has revealed that numerous scleractinian and gorgonian species possess a bacterial core microbiome, i.e., they consistently associate with certain bacteria regardless of space and time. Black corals, however, seem to largely lack this feature, as no core microbiome has been detected in *A. subpinnata* here or the other species studied in-depth, *L. glaberrima* (Dannenberg, 2016). The high level of microbiome flexibility in *A. subpinnata* may explain this lack of a bacterial ‘core microbiome.’ In agreement with Ziegler et al. (2019), we believe that, due to the differences in the degree of microbiome flexibility among coral species, elucidating a universal coral core microbiome (Ainsworth et al., 2015; Hernandez-Agreda et al., 2017) is difficult. However, recent papers have described strong signals of phylosymbiosis in both tropical reef-building hexacorals (Pollock et al., 2018) and the distantly related Mediterranean gorgonian octocorals (van de Water et al., 2018b). Phylosymbiosis has been defined as “microbial community relationships that recapitulate the phylogeny of their host” (Brucker and Bordenstein, 2013; Lim and Bordenstein, 2020). It is therefore surprising to see that the Antipatharia-branch of the Hexacorallia may not fit in the pattern of phylosymbiosis in corals as it appears to lack close relationships with its microbiota. Phylosymbiosis has often been considered to arise from long-term associations between a host and its microbes (e.g., co-diversification in scleractinian and gorgonian corals, Pollock et al., 2018; van de Water et al., 2018b). However, a recent study on *Drosophila melanogaster* found that phylosymbiosis may also be driven by short-term changes in the microbiota (Rudman et al., 2019). Consequently, Lim and Bordenstein (2020) suggest that microbial communities are not passive agents of phylosymbiosis, but may have the potential to induce genomic changes in the host that could impact establishment, maintenance or breakdown of phylosymbiosis. Whether a breakdown of phylosymbiosis in the Antipatharia occurred during the evolution of the Hexacorallia or phylosymbiosis arose multiple times during coral evolution remains an open question.

## Conclusion

The composition of the bacterial communities of black corals is more similar to the microbial community associated with the reef-building scleractinian corals than the structurally similar, but more distantly related, and sympatric gorgonian corals. The potential lack of a core microbiome and major spatial and temporal differences observed shows that environmental factors largely determine the compositional differences of the black coral-associated microbial communities. However, whether the functioning of the microbiota has changed or that there is functional redundancy remains to be assessed through functional and metagenomics studies. This may suggest that black corals, such as *A. subpinnata*, have limited microbiome regulatory capacities. Yet, microbiome flexibility may also allow these corals to tailor their microbiota to local, and potentially changing, environmental conditions by selecting the most beneficial microbes from the surrounding environment. This could thus be another strategy that may have contributed to the black corals’ success in colonizing the mesophotic zone and deep sea.

## Data Availability Statement

The datasets generated for this study can be found in the NCBI Sequence Read Archive: PRJNA506661.

## Author Contributions

JW, MC, MB, and CF-P designed the study. MC, FE, and MB collected and processed the samples. MC performed the DNA extractions. JW analyzed the sequencing data. JW, MC, MB, and CF-P wrote the manuscript. All the authors reviewed, edited and approved the final manuscript.

## Conflict of Interest

The authors declare that the research was conducted in the absence of any commercial or financial relationships that could be construed as a potential conflict of interest.

## Footnotes

1 https://www.drive5.com/usearch/

2 https://www.genome.jp/kegg/
