# Supplementary material for: Local Conditions Influence the Prokaryotic Communities Associated With the Mesophotic Black Coral Antipathella subpinnata
Source: Front Microbiol. 2020 Oct 6;11:537813. doi: 10.3389/fmicb.2020.537813 (PMC7573217; doi:10.3389/fmicb.2020.537813)
Supplement: Supplementary file 1 [file Data_Sheet_1.ZIP › Suppl File S8 - NB-GLM results functional groups.docx]

#################################################################################

Outcomes of statistical analysis of abundances in functional groups using negative binomial generalized linear models

#################################################################################

By Location (data = AsubM -- Antipathella subpinnata samples collected at three locations in May 2017):

#################################################################################

glm.nb(formula = DMSPcleavage ~ Location, data = AsubM, init.theta = 1.310916704, link = log)

Deviance Residuals:

Min 1Q Median 3Q Max

-2.1400 -0.8489 -0.4481 0.3916 2.0506

Estimate Std. Error z value Pr(>|z|)

(Intercept) 8.0876 0.2248 35.970 < 2e-16 ***

factor.glmPortofino 0.1612 0.3278 0.492 0.62273

factor.glmSavona -0.8319 0.2975 -2.796 0.00517 **

Simultaneous Tests for General Linear Hypotheses

Multiple Comparisons of Means: Tukey Contrasts

Estimate Std. Error z value Pr(>|z|)

Portofino - Bordighera == 0 0.1612 0.3278 0.492 0.87494

Savona - Bordighera == 0 -0.8319 0.2975 -2.796 0.01438 *

Savona - Portofino == 0 -0.9932 0.3079 -3.225 0.00375 **

(Adjusted p values reported -- single-step method)

---------------------------------------------------------------------------------

---------------------------------------------------------------------------------

glm.nb(formula = DMSPDemethylation ~ Location, data = AsubM, init.theta = 3.620103178, link = log)

Deviance Residuals:

Min 1Q Median 3Q Max

-1.9425 -1.1579 0.2633 0.7448 1.5127

Estimate Std. Error z value Pr(>|z|)

(Intercept) 8.3809 0.1753 47.818 < 2e-16 ***

factor.glmPortofino -1.6607 0.2557 -6.494 8.36e-11 ***

factor.glmSavona -3.3359 0.2330 -14.319 < 2e-16 ***

Simultaneous Tests for General Linear Hypotheses

Multiple Comparisons of Means: Tukey Contrasts

Estimate Std. Error z value Pr(>|z|)

Portofino - Bordighera == 0 -1.6607 0.2557 -6.494 <1e-09 ***

Savona - Bordighera == 0 -3.3359 0.2330 -14.319 <1e-09 ***

Savona - Portofino == 0 -1.6752 0.2413 -6.942 <1e-09 ***

(Adjusted p values reported -- single-step method)

---------------------------------------------------------------------------------

---------------------------------------------------------------------------------

glm.nb(formula = SulphurOxidation ~ Location, data = AsubM, init.theta = 5.274529693, link = log)

Deviance Residuals:

Min 1Q Median 3Q Max

-2.3862 -0.7629 0.0159 0.7926 1.1694

Coefficients:

Estimate Std. Error z value Pr(>|z|)

(Intercept) 8.4292 0.1452 58.043 < 2e-16 ***

factor.glmPortofino -1.4978 0.2119 -7.068 1.57e-12 ***

factor.glmSavona -3.0355 0.1930 -15.724 < 2e-16 ***

Simultaneous Tests for General Linear Hypotheses

Multiple Comparisons of Means: Tukey Contrasts

Estimate Std. Error z value Pr(>|z|)

Portofino - Bordighera == 0 -1.4978 0.2119 -7.068 <1e-10 ***

Savona - Bordighera == 0 -3.0355 0.1930 -15.724 <1e-10 ***

Savona - Portofino == 0 -1.5377 0.2000 -7.689 <1e-10 ***

(Adjusted p values reported -- single-step method)

---------------------------------------------------------------------------------

---------------------------------------------------------------------------------

Call:

glm.nb(formula = SulfateReduction ~ Location, data = AsubM, init.theta =

0.3658548806, link = log)

Deviance Residuals:

Min 1Q Median 3Q Max

-2.00001 -1.60344 -0.35811 0.00416 1.38888

Estimate Std. Error z value Pr(>|z|)

(Intercept) 4.4569 0.5523 8.070 7.01e-16 ***

factor.glmPortofino 0.5050 0.8047 0.628 0.53025

factor.glmSavona -1.9790 0.7347 -2.694 0.00707 **

Simultaneous Tests for General Linear Hypotheses

Multiple Comparisons of Means: Tukey Contrasts

Estimate Std. Error z value Pr(>|z|)

Portofino - Bordighera == 0 0.5050 0.8047 0.628 0.80468

Savona - Bordighera == 0 -1.9790 0.7347 -2.694 0.01927 *

Savona - Portofino == 0 -2.4840 0.7598 -3.269 0.00306 **

(Adjusted p values reported -- single-step method)

---------------------------------------------------------------------------------

---------------------------------------------------------------------------------

Call:

glm.nb(formula = NitrogenFixation ~ Location, data = AsubM, init.theta = 2.915223796, link = log)

Deviance Residuals:

Min 1Q Median 3Q Max

-2.72676 -0.76929 -0.00672 0.35830 1.74778

Estimate Std. Error z value Pr(>|z|)

(Intercept) 6.0466 0.1959 30.866 < 2e-16 ***

factor.glmPortofino -0.5216 0.2859 -1.824 0.0681 .

factor.glmSavona -1.9074 0.2613 -7.299 2.9e-13 ***

Simultaneous Tests for General Linear Hypotheses

Multiple Comparisons of Means: Tukey Contrasts

Estimate Std. Error z value Pr(>|z|)

Portofino - Bordighera == 0 -0.5216 0.2859 -1.824 0.161

Savona - Bordighera == 0 -1.9074 0.2613 -7.299 <1e-04 ***

Savona - Portofino == 0 -1.3858 0.2707 -5.119 <1e-04 ***

(Adjusted p values reported -- single-step method)

---------------------------------------------------------------------------------

---------------------------------------------------------------------------------

Call:

glm.nb(formula = Ammonification ~ Location, data = AsubM, init.theta = 3.418775408, link = log)

Deviance Residuals:

Min 1Q Median 3Q Max

-1.5119 -1.0149 -0.3961 0.5575 1.9585

Estimate Std. Error z value Pr(>|z|)

(Intercept) 8.7090 0.1803 48.295 <2e-16 ***

factor.glmPortofino -0.3985 0.2629 -1.516 0.13

factor.glmSavona -2.2905 0.2388 -9.591 <2e-16 ***

Simultaneous Tests for General Linear Hypotheses

Multiple Comparisons of Means: Tukey Contrasts

Estimate Std. Error z value Pr(>|z|)

Portofino - Bordighera == 0 -0.3985 0.2629 -1.516 0.283

Savona - Bordighera == 0 -2.2905 0.2388 -9.591 <1e-04 ***

Savona - Portofino == 0 -1.8920 0.2472 -7.654 <1e-04 ***

(Adjusted p values reported -- single-step method)

---------------------------------------------------------------------------------

---------------------------------------------------------------------------------

Call:

glm.nb(formula = Nitrification ~ Location, data = AsubM, init.theta = 0.325031132,link = log)

Deviance Residuals:

Min 1Q Median 3Q Max

-1.5107 -1.3444 -0.5002 0.2217 1.0307

Estimate Std. Error z value Pr(>|z|)

(Intercept) 2.3567 0.5936 3.970 7.19e-05 ***

factor.glmPortofino 1.8100 0.8596 2.106 0.0352 *

factor.glmSavona -0.7640 0.7910 -0.966 0.3341

Simultaneous Tests for General Linear Hypotheses

Multiple Comparisons of Means: Tukey Contrasts

Estimate Std. Error z value Pr(>|z|)

Portofino - Bordighera == 0 1.8100 0.8596 2.106 0.08846 .

Savona - Bordighera == 0 -0.7640 0.7910 -0.966 0.59813

Savona - Portofino == 0 -2.5740 0.8123 -3.169 0.00428 **

(Adjusted p values reported -- single-step method)

---------------------------------------------------------------------------------

---------------------------------------------------------------------------------

Call:

glm.nb(formula = Denitrification ~ Location, data = AsubM, init.theta = 1.785623282, link = log)

Deviance Residuals:

Min 1Q Median 3Q Max

-2.5776 -0.9678 -0.4629 0.4355 2.2210

Estimate Std. Error z value Pr(>|z|)

(Intercept) 8.2964 0.2495 33.251 < 2e-16 ***

factor.glmPortofino -1.5134 0.3639 -4.159 3.19e-05 ***

factor.glmSavona -4.1612 0.3320 -12.532 < 2e-16 ***

Simultaneous Tests for General Linear Hypotheses

Multiple Comparisons of Means: Tukey Contrasts

Estimate Std. Error z value Pr(>|z|)

Portofino - Bordighera == 0 -1.5134 0.3639 -4.159 0.000117 ***

Savona - Bordighera == 0 -4.1612 0.3320 -12.532 < 1e-04 ***

Savona - Portofino == 0 -2.6479 0.3437 -7.703 < 1e-04 ***

(Adjusted p values reported -- single-step method)

#################################################################################

By time point (data = AsubT -- Antipathella subpinnata samples collected at Portofino location in November 2016 and May 2017)

#################################################################################

Call:

glm.nb(formula = DMSPcleavage ~ Time, data = AsubT, init.theta = 3.504343564,

link = log)

Deviance Residuals:

Min 1Q Median 3Q Max

-1.6298 -0.8838 -0.2942 0.2916 1.9819

Estimate Std. Error z value Pr(>|z|)

(Intercept) 8.2489 0.1890 43.656 <2e-16 ***

factor.glmNovember -0.2629 0.2535 -1.037 0.3

---------------------------------------------------------------------------------

---------------------------------------------------------------------------------

Call:

glm.nb(formula = DMSPdemethylation ~ Time, data = AsubT, init.theta = 2.114255144,link = log)

Deviance Residuals:

Min 1Q Median 3Q Max

-2.0440 -1.2382 -0.2782 0.5560 1.3388

Estimate Std. Error z value Pr(>|z|)

(Intercept) 6.7202 0.2435 27.603 <2e-16 ***

factor.glmNovember 0.1168 0.3266 0.358 0.721

---------------------------------------------------------------------------------

---------------------------------------------------------------------------------

Call:

glm.nb(formula = SulphurOxidation ~ Time, data = AsubT, init.theta = 2.91397648, link = log)

Deviance Residuals:

Min 1Q Median 3Q Max

-1.7590 -1.0982 -0.1123 0.5641 1.5134

Estimate Std. Error z value Pr(>|z|)

(Intercept) 6.93135 0.20741 33.42 <2e-16 ***

factor.glmNovember -0.04717 0.27828 -0.17 0.865

---------------------------------------------------------------------------------

---------------------------------------------------------------------------------

Call:

glm.nb(formula = SulfateReduction ~ Time, data = AsubT, init.theta = 1.757986552, link = log)

Deviance Residuals:

Min 1Q Median 3Q Max

-2.3546 -0.7626 -0.4264 0.4831 1.5604

Estimate Std. Error z value Pr(>|z|)

(Intercept) 4.961970 0.268289 18.495 <2e-16 ***

factor.glmNovember -0.001226 0.359949 -0.003 0.997

---------------------------------------------------------------------------------

---------------------------------------------------------------------------------

Call:

glm.nb(formula = NitrogenFixation ~ Time, data = AsubT, init.theta = 2.514414836, link = log)

Deviance Residuals:

Min 1Q Median 3Q Max

-2.0638 -0.7466 -0.2476 0.1793 2.6136

Estimate Std. Error z value Pr(>|z|)

(Intercept) 5.52495 0.22408 24.656 <2e-16 ***

factor.glmNovember -0.06197 0.30068 -0.206 0.837

---------------------------------------------------------------------------------

---------------------------------------------------------------------------------

Call:

glm.nb(formula = Nitrification ~ Time, data = AsubT, init.theta = 0.8984062144, link = log)

Deviance Residuals:

Min 1Q Median 3Q Max

-2.6427 -0.9419 -0.3084 0.2339 1.8628

Estimate Std. Error z value Pr(>|z|)

(Intercept) 4.1667 0.3756 11.093 <2e-16 ***

factor.glmNovember -0.4078 0.5047 -0.808 0.419

---------------------------------------------------------------------------------

---------------------------------------------------------------------------------

Call:

glm.nb(formula = Ammonification ~ Time, data = AsubT, init.theta = 3.357411181,

link = log)

Deviance Residuals:

Min 1Q Median 3Q Max

-1.5837 -0.9940 -0.3895 0.6266 1.9409

Estimate Std. Error z value Pr(>|z|)

(Intercept) 8.310 0.193 43.052 < 2e-16 ***

factor.glmNovember -0.867 0.259 -3.347 0.000817 ***

---------------------------------------------------------------------------------

---------------------------------------------------------------------------------

Call:

glm.nb(formula = Denitrification ~ Time, data = AsubT, init.theta = 1.074714287,

link = log)

Deviance Residuals:

Min 1Q Median 3Q Max

-1.8965 -0.9327 -0.4597 0.4586 1.7236

Estimate Std. Error z value Pr(>|z|)

(Intercept) 6.7830 0.3413 19.877 <2e-16 ***

factor.glmNovember -0.2822 0.4579 -0.616 0.538
