# Supplementary material for: Local Conditions Influence the Prokaryotic Communities Associated With the Mesophotic Black Coral Antipathella subpinnata
Source: Front Microbiol. 2020 Oct 6;11:537813. doi: 10.3389/fmicb.2020.537813 (PMC7573217; doi:10.3389/fmicb.2020.537813)
Supplement: Supplementary file 1 [file Data_Sheet_1.ZIP › Suppl File S1 - Contaminant sequence identification - decontam outcomes.docx]

**Distribution of decontam scores – coral samples**


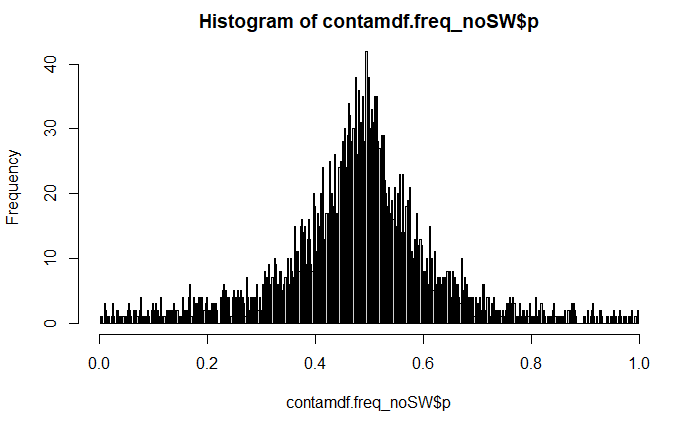


**Potential contaminant OTUs (cut-off decontam score: p < 0.05)**

|  | freq | prev | p.freq | p.prev | p | contaminant |
| --- | --- | --- | --- | --- | --- | --- |
| Otu9512 | 1.09E-05 | 2 | 0.003514 | NA | 0.003514 | TRUE |
| Otu4509 | 1.43E-05 | 2 | 0.004237 | NA | 0.004237 | TRUE |
| Otu4064 | 2.72E-05 | 2 | 0.010193 | NA | 0.010193 | TRUE |
| Otu3797 | 2.95E-05 | 3 | 0.011268 | NA | 0.011268 | TRUE |
| Otu3391 | 2.65E-05 | 3 | 0.011732 | NA | 0.011732 | TRUE |
| Otu6157 | 1.86E-05 | 2 | 0.013074 | NA | 0.013074 | TRUE |
| Otu10386 | 9.02E-06 | 2 | 0.013735 | NA | 0.013735 | TRUE |
| Otu4885 | 2.63E-05 | 2 | 0.016793 | NA | 0.016793 | TRUE |
| Otu5829 | 2.92E-05 | 3 | 0.018736 | NA | 0.018736 | TRUE |
| Otu3691 | 3.85E-05 | 3 | 0.022022 | NA | 0.022022 | TRUE |
| Otu10209 | 3.94E-06 | 2 | 0.024272 | NA | 0.024272 | TRUE |
| Otu1222 | 0.000108 | 3 | 0.024532 | NA | 0.024532 | TRUE |
| Otu4851 | 1.75E-05 | 2 | 0.025628 | NA | 0.025628 | TRUE |
| Otu700 | 1.56E-06 | 2 | 0.027761 | NA | 0.027761 | TRUE |
| Otu2531 | 3.44E-05 | 3 | 0.032255 | NA | 0.032255 | TRUE |
| Otu7511 | 1.19E-05 | 2 | 0.033655 | NA | 0.033655 | TRUE |
| Otu4704 | 2.07E-05 | 2 | 0.03452 | NA | 0.03452 | TRUE |
| Otu5097 | 1.69E-05 | 2 | 0.035002 | NA | 0.035002 | TRUE |
| Otu4853 | 3.22E-05 | 3 | 0.037713 | NA | 0.037713 | TRUE |
| Otu7485 | 1.12E-05 | 2 | 0.039947 | NA | 0.039947 | TRUE |
| Otu1025 | 2.97E-05 | 2 | 0.040557 | NA | 0.040557 | TRUE |
| Otu2691 | 1.73E-05 | 2 | 0.043371 | NA | 0.043371 | TRUE |
| Otu3470 | 2.37E-05 | 2 | 0.045735 | NA | 0.045735 | TRUE |
| Otu9321 | 9.76E-06 | 2 | 0.049993 | NA | 0.049993 | TRUE |

- ‘prev’ indicates the prevalence (i.e. in how many samples the OTU is observed)
- ‘p’ is the decontam score

**Distribution of decontam scores – seawater samples**
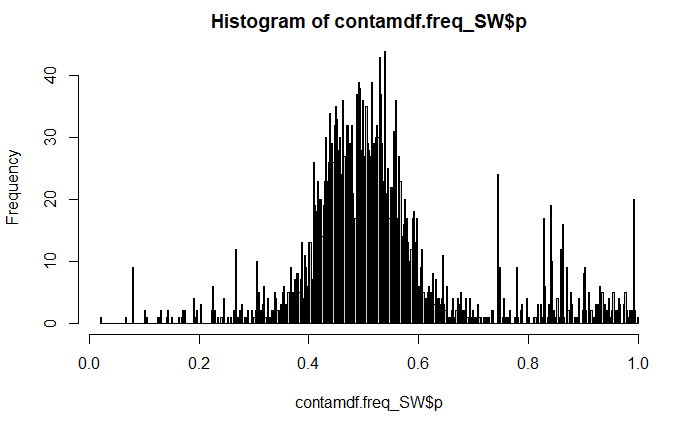


**Potential contaminant OTUs (cut-off decontam score: p < 0.05)**

|  | freq | prev | p.freq | p.prev | p | contaminant |
| --- | --- | --- | --- | --- | --- | --- |
| Otu15157 | 5.15E-06 | 2 | 0.020945 | NA | 0.020945 | TRUE |

- ‘prev’ indicates the prevalence (i.e. in how many samples the OTU is observed)
- ‘p’ is the decontam score
